# Comparison of swimming capacity and energetics of migratory European eel (*Anguilla anguilla*) and New Zealand short-finned eel (*A. australis*)

**DOI:** 10.3389/fphys.2015.00256

**Published:** 2015-09-17

**Authors:** Christian Tudorache, Erik Burgerhout, Sebastiaan Brittijn, Guido van den Thillart

**Affiliations:** ^1^Department for Animal Sciences and Health, Institute Biology Leiden, Leiden UniversityLeiden, Netherlands; ^2^NewCatch B.V.Leiden, Netherlands

**Keywords:** ecology, physiology, respirometry, biometry, fish

## Abstract

The spawning migration of the European eel (*Anguilla anguilla*) can cover more than 6000 km, while that of the New Zealand short-finned eel (*A. australis*) is assumed to be approximately 3000 km. Since these species are expected to show adaptive traits to such an important lifetime event, we hypothesized differences in swimming capacity and energetics as a response to this adaptation. In an experimental swimming respirometer set-up, critical swimming speed (U_crit_), optimal swimming speed (U_opt_), mass specific oxygen consumption rate (ṀO_2_), standard metabolic rate (SMR), active metabolic rate at U_crit_ (AMR_crit_) and at U_opt_ (AMR_opt_), the minimum cost of transport at U_opt_ (COT_min_), and the scope for activity, were assessed and compared between the species. With a similar body length and mass, European eels showed ca. 25% higher values for both U_crit_ and U_opt_, and 23% lower values for COT_min_, compared to New Zealand short-finned eels. However, SMR, AMR_crit_, AMR_opt_, and scope for activity did not differ between the species, indicating very similar swimming physiology traits. This study discusses physiological aspects of long distance migration and provides recommendations for (a) swimming respirometry in anguilliform fish, and (b) telemetry research using externally attached pop-up tags.

## Introduction

Fish species have evolved strategies, which enable them to use different types of habitats during their life cycle (for reviews see Lucas and Baras, 2001; Tesch, 2003). Facultative catadromic species such as anguillid eels (Tesch, 2003; Aoyama, 2009; Jellyman et al., 2009) use marine habitats for reproduction and freshwater or brackish habitats for growth and differentiation. During an extremely complex life cycle, anguillid eels develop through oceanic larval (leptocephali) and juvenile stages (glass eels), and many years of growth and differentiation in freshwater or brackish habitats, into a migratory “silver” stage. At the end of their lives, these silver eels perform an oceanic reproductive migration to their spawning grounds where they spawn and subsequently die (semelparity; Tesch, 2003).

The location of the spawning site of the European eel (*Anguilla anguilla*) is still a mystery but early studies strongly indicate the Southwest Sargasso Sea based on the location of the smallest larvae found (Schmidt, 1923, 1925). Since the start of the spawning migration occurs in autumn and the first glass eels arrive at European coasts in spring it is suggested that migrating silver eels cover 6000 km or more within 6 months to reach their spawning grounds in April (Tesch, 2003). The newly hatched leptocephalus larvae subsequently drift with the golf stream for a year until they are recruited as glass eels at the European coast during the next spring (Schmidt, 1923; Tesch, 2003). However, no adult spawning eels have been observed in the Sargasso Sea to date, nor have eggs been found.

Equally mysterious is the spawning migration of the New Zealand short-finned eel (*A. australis*). Only a few leptocephalus larvae have been found in an area located Northwest of the Fiji Islands (Castle, 1963). Based on these findings, Castle (1963) suggests a potential spawning site between the Fiji Islands and Tahiti (170°W by 18°S), while Jellyman (1987) argues that a more Northern area (between 150 and 170°W by 5 and 15°S) was likely, based on literature data of oceanography, body size, time of larval recruitment, otolith microstructure, and gonadal development for migrating silver eels. The linear migration distance from the most distant area of distribution, i.e., Southern Victoria and Tasmania (Dijkstra and Jellyman, 1999) to these potential spawning sites would amount to ca. 3000 km.

Assuming similar migration behavior and strategies for both species during spawning migration, but greatly different distances, the total energy expenditure would be higher for European than for New Zealand short-finned eels. Therefore, we hypothesize that swimming capacity and energetics correspond with migration distances, resulting in an overall higher swimming capacity and a lower energy expenditure over distance swum for European than for New Zealand short-finned eels.

## Materials and methods

Wild female silver European eels (*A. anguilla, N* = 7; body mass and body length, see Table 1; silver index 4–5, Durif et al., 2005) were caught by means of fykes in Lake Veerse, The Netherlands, at the end of October 2011, and transported to our laboratory in Leiden in large barrels with a small amount of water.

**Table 1:** **Data on biometrics (body length, mass, maximum cross sectional area), swimming capacity (critical swimming speed, U_crit_), and swimming energetics of European (EU) and New Zealand short-finned eels (NZ)**.

	**EU**	**NZ**	***p*-value**
Body length (mm)	773 ± 37	746 ± 25	0.56
Body mass (g)	905.67 ± 145.29(a)	808.69 ± 79.09(a)	0.57
Maximum cross sectional area (mm^2^)	167 ± 4	173 ± 4	0.31
SMR(mgO_2_kg^−1^h^−1^)	45.65 ± 2.12	50.52 ± 3.11	0.22
U_crit_(m s^−1^)	0.94 ± 0.02	0.74 ± 0.03	**0.0002**
U_opt_(m s^−1^)	0.64 ± 0.03	0.51 ± 0.02	**0.004**
COT_min_(mgO_2_kg^−1^ m^−1^)	54.48 ± 2.13	67.91 ± 2.17	**0.0008**
AMR_opt_(mgO_2_kg^−1^h^−1^)	125.55 ± 5.76	124.95 ± 4.5	0.94
AMR_crit_(mgO_2_kg^−1^h^−1^)	206.44 ± 11.41	197.26 ± 9.49	0.55
Scope for activity (mgO_2_kg^−1^h^−1^)	160.25 ± 11.27	151.29 ± 9.58	0.56

**Figure 1:**
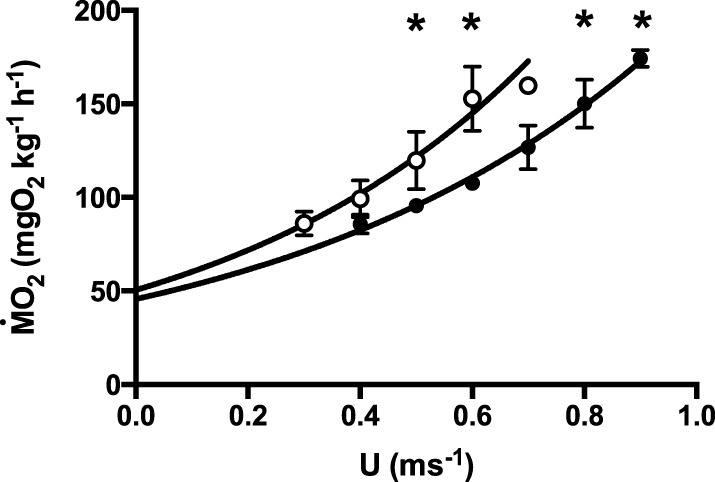
**Relative oxygen consumption rate (ṀO_2_, mgO_2_ kg^−1^ h^−1^) as a function of swimming speed (U, m s^−1^) for EU (full circles) and NZ (empty circles)**. Exponential function ṀO_2_ = SMRe^cU^, with SMR the standard metabolic rate, e Euler's constant and c constant, and U swimming speed (for values see Table 1). Data are mean ± SE, *r*^2^ = 8.9 ± 2.2. Curve fitting data are given in Table 1. ^*^indicates significant difference between species at the respective speed: *t*-test, *p* < 0.05, *N* = 7.

*Oxygen consumption rate (ṀO_2_, mgO_2_kg^−1^ h^−1^, see Figure 1) was expressed as a exponential function of swimming speed (U, m s^−1^): ṀO_2_ = SMRe^cU^, with SMR the standard metabolic rate, e Euler's constant, and c constant. From this function, optimal swimming speed (U_opt_, m s^−1^), minimum Cost of Transport (COT_min_, mgO_2_ kg^−1^ km^−1^), active metabolic rate at U_opt_ (AMR_opt_, mgO_2_kg^−1^ h^−1^), and at U_crit_, the critical swimming speed (AMR_crit_) and the scope for activity (mgO_2_kg^−1^h^−1^) were derived. Values are mean ± SE, significance was accepted at p < 0.05 (bold), t-test, N = 7*.

Wild female silver New Zealand short-finned eels (*A. australis, N* = 7; body mass and body length, see Table 1, migratory pre-reproductive stage, Lokman et al., 1998) were caught by means of fykes in Lake Ellesmere, Christchurch, New Zealand, in March 2011, and transported to The Netherlands in aerated plastic bags with a small amount of water, fitted into cooled polystyrene boxes (5–10°C), with the journey lasting for 2.5 days.

After arrival in the laboratory in Leiden, The Netherlands, eels were acclimated for ca. 2 weeks in a 4000 L recirculation system, supplied with natural seawater (28 ± 1 ppt) at 18 ± 1°C (water and air temperature) with an air saturation of 75–85%, situated in a climate cell for constant conditions. Fish were kept under dimmed light to reduce stress before and during the trials. Migratory eels cease feeding, so they were not fed during the entire period of time. Since both species originated from brackish water bodies, and (pre) migrating eels are very adaptive to rapid changes in salinity (Tesch, 2003), adaptation to the higher salinity of the holding facility was not assumed to be stressful. Indeed, all eels responded very well to transport and transition to seawater, without symptoms of discomfort or stress, and the animals appeared lively and agile. The eels kept their silver stage during the entire experimental period.

### Preparation and acclimation

For preparatory handling before swimming trials i.e., measurement of body mass, length, height, and width for calculation of cross sectional areas, eels were anesthetized with clove oil, dissolved in 96% ethanol at a ratio of 1:10, which was dosed 1 ml in 1 l seawater. All eels were completely unresponsive under anesthesia when measured and weighed. Biometric data show no significant differences between species in body length or body mass (Table 1).

Subsequently, the eels were transferred to 14 identical 127 l Blazka-type swimming tunnel (swim section length: 1150 mm, diameter: 190 mm, identical flow profiles; described in van den Thillart et al., 2004) connected to the recirculation system, in which the swimming trials (seven European and seven New Zealand short finned eels) were performed simultaneously in order to avoid time related effects.

Upon transfer to the swimming tunnels, the eels recovered after 1–5 min, showing routine activity. The animals were allowed to fully recover over a period of 16–24 h, which is considered sufficient for swimming fish (e.g., Lee et al., 2003; Svendsen et al., 2010). The water speed was set at 0.1 m s^−1^, at which they would remain coiled up against the rear grid of the swimming tunnels. This low velocity ensured an equal distribution of oxygenated water in the tunnel (Burgerhout et al., 2011; Methling et al., 2011; Tudorache et al., 2014). The tunnels were covered with a black plastic sheet in order to reduce stress due to visual disturbance.

### Swimming trials

Swimming capacity was estimated by critical swimming speed (U_crit_), the maximum sustained swimming speed (Brett, 1964), and mass specific oxygen consumption rate (ṀO_2_) and derived energetic values provided information about energy use during long term migration.

At water speeds of a minimum of 0.4 m s^−1^ for European and 0.3 m s^−1^ for New Zealand short finned eels, fish would orientate themselves against the stream and hold position in the tunnel using a regular swimming mode, characterized by a steady anterior position, visually uniform tail beat frequency and amplitude. Oxygen consumption rate data below these swimming velocities were not included in the analysis.

First, the animals were subjected to a U_crit_-test. Water velocity was increased in increments of 0.1 m s^−1^ at intervals of 20 min (Methling et al., 2011) until the fish fatigued, i.e., refused to swim and was flushed against the downstream grid of the tunnel where it remained for at least 20 s. After fatigue, fish were allowed to recover at a water speed of 0.1 m s^−1^ for 16–24 h.

Subsequently, for the calculation of ṀO_2_, eels were subjected to a series of swimming speeds ranging from 0.3 to 0.9 m s^−1^ with 0.1 m s^−1^ increments and 60 min intervals. Flushing with oxygenated water from the recirculation system occurred during the first 30 min (air saturation 82.7 ± 5.3%), and oxygen concentration ([O_2_]) was measured during the last 30 min of each swimming period, to allow a steady measurement without the possible effects of a fish stressed by previous changes in swimming velocity. [O_2_] was measured with a galvanic oxygen electrode (type Inpro 6415, Mettler Toledo, The Netherlands) and logged with a HP 34970A multichannel logger and controller, connected to two 40-channel multiplexers (34907 and 34901 A). Data were sampled at a rate of 0.1 Hz. The air saturation never fell below 70% during the test and water temperature was constantly 18 ± 1°C. Background oxygen consumption rate of the system was previously measured (van den Thillart et al., 2004) and < 2% h^−1^. After trials, eels were removed and reused in other studies.

### Data analysis

U_crit_ was calculated according to the equation:

$${\text{U}}_{\text{crit}}{=\text{U}}_{\text{i}}\;+\;[\Delta \text{U }{(}_{\text{Ti}}\;\Delta {\text{T}}^{-1})],$$

where U_*i*_ is the highest velocity maintained for the entire 20 min interval, ΔU is the velocity increment (0.1 m s^−1^), T_*i*_ is the duration of the final (fatigue) step and ΔT is the time interval (20 min; Brett, 1964).

ṀO_2_ (mgO_2_kg^−1^ h^−1^) was fitted as a function of swimming speed (U) to the exponential equation:

$$\dot{\text{M}}{\text{O}}_{2}\;=\text{ SMR }{\text{e}}^{\text{cU}},$$

with SMR, the standard metabolic rate; e, Euler's constant; and c, being constant. The optimal swimming speed, (U_opt_), i.e., the swimming speed with minimum energy consumption, was calculated from this exponential function by

$${\text{U}}_{\text{opt}}\;=\;1/\text{c}$$

and the minimum cost of transport (COT_min_ in mgO_2_kg^−1^ km^−1^), i.e., the lowest cost over distance, swum at U_opt_, was calculated by

$${\text{COT}}_{\text{min}}\;=\;\frac{\dot{\text{M}}{\text{O}}_{2}\left({\text{U}}_{\text{opt}}\right)}{{\text{U}}_{\text{opt}}}$$

(Petterson and Hedenström, 2000).

Resulting swimming speeds (U_crit_, U_opt_) and other calculated parameters were corrected for the solid blocking effect according to Bell and Terhune (1970):

$${\text{U}}_{\text{F}}\;=\;{\text{U}}_{\text{T}}\left(1+\varepsilon_{\text{S}}\right)$$

with U_F_ the corrected speed, U_T_ the original speed, and ε_S_ the fractional error quotient:

$$\varepsilon_{\text{S}}\;=\;\tau \lambda {{(}_{\text{AO}}{/\text{A}}_{\text{T}})}^{3/2}$$

with τ a dimensionless factor depending on flume cross-sectional shape, λ a shape factor for the test object (0.5), A_O_ the maximum cross-sectional area of the fish, and A_T_ the cross-sectional area of swimming section (Bell and Terhune, 1970).

The critical (AMR_crit_) and optimal active metabolic rate (AMR_opt_) are the ṀO_2_ at U_*crit*_ and at U_opt_, respectively. The scope for activity is the difference between SMR and AMR_crit_.

### Statistics

Data (U_crit_, U_opt_, body length, body mass, maximum cross sectional area, SMR, COT_min_, AMR_opt_, AMR_crit_, Scope for activity, ṀO_2_) and residuals were tested for normal distribution with a Kolmogorov-Smirnoff test; after confirmation (*p* < 0.05, *N* = 7), they were compared between eel species using a student-*t*-test (SigmaPlot v. 11, Systat systems inc. USA). In order to test for an effect of the variation of body mass on ṀO_2_, and body length on U_crit_ and U_opt_, respectively, an ANCOVA was performed to compare absolute values of metabolic rate with mass and size as corresponding covariates in the analyses and to account for differences in mass and size among individuals and between species. Significance was determined in all cases at *p* < 0.05. Data are given as mean ± SE.

### Ethics statement

This study complied with the Dutch Law on Animal Experiments and was approved by the Animal Ethical Committee of Leiden University (DEC# 10231). All measurement was performed under clove oil anesthesia, and all efforts were made to minimize suffering and reduce the number of animals used.

## Results

After correction for the solid blocking effect, critical swimming speed (U_crit_, Table 1) was ca. 25% higher in European than in New Zealand short-finned eels (*p* < 0.01), as was optimal swimming speed (U_opt_; *p* < 0.01, Table 1). Mass specific oxygen consumption rates (ṀO_2_; Figure 1) were lower in European than in New Zealand short-finned eels at all speeds above 0.5 m s^−1^ (*p* < 0.01; Figure 1), as were minimum cost of transport (COT_min_) values (*p* < 0.01, Table 1). However, other energetic values (Table 1) such as the extrapolated standard metabolic rate (SMR), the active metabolic rate at U_opt_ (AMR_opt_) and at U_crit_ (AMR_crit_), and the scope for activity, were not significantly different between species (*p* > 0.05). ANCOVA analysis confirmed that differences in absolute oxygen consumption rates and swimming velocities (U_crit_ and U_opt_) did not depend on differences in body mass between the species (*p*-values for EU and NZ, respectively: ṀO_2_ at 0.5 m s^−1^ 0.13 and 0.27; ṀO_2_ at 0.6 m s^−1^ 0.14 and 0.23; U_crit_ 0.21 and 0.32; U_opt_ 0.13 and 0.22).

## Discussion

To our knowledge, this is the first direct estimate of swimming capacity and energetics of New Zealand short-finned eel (*A. australis*) and its comparison with European eel (*A. anguilla*). The distance of their spawning migrations differs greatly, with 6000 and 3000 km, respectively. Based on a possible evolutionary adaptation to strongly different migration distances between the species, we hypothesized differences in swimming capacity and energetic parameters. Indeed, the results show that regardless the similarity in size and mass of the tested animals, the critical (U_crit_) and the optimal swimming speed (U_opt_) were significantly higher in European than in New Zealand short-finned eels, indicating an increased swimming capacity. However, when extrapolating U_crit_ or U_opt_ to oxygen consumption rate (ṀO_2_), the resulting critical or optimal active metabolic rates (AMR_crit_, AMR_opt_) were similar between the species. These results suggest no difference in maximum and minimum aerobic expenditure rates and therefore similar energetic profiles between the species.

The observed differences in swimming capacity (U_crit_ and U_opt_), contrasting the similar energetic profiles (AMR_crit_ and AMR_opt_), may be explained in terms of muscle power output. Altringham and Ellerby (1999) showed that muscle function can vary among species. For example, it appears that many species have different distributions of slow, aerobic muscles, with a decrease of muscle twitch speed from anterior to posterior along the body axis (Altringham et al., 1993; Davies and Johnston, 1993; Rome et al., 1993; Davies et al., 1995; Altringham and Block, 1997). However, Ellerby et al. (2001) showed that the change in isometric properties of slow muscles along axial location is less marked in eels than in most other species. Time from stimulus to peak force does not change significantly with axial position (Ellerby et al., 2001). Since anguilliform swimmers show uniform muscle kinetics along the body axis, swimming with different tail beat amplitude or frequency, and therefore stride length (the distance covered per tail beat cycle; Videler and Wardle, 1991), can possibly account for the differences in swimming capacity observed between European and New Zealand eels. Future research should clarify if swimming kinematics is accountable for the observed differences in swimming capacity and energetics as related to red muscle power output.

Similar to AMR_crit_ and AMR_opt_, the standard metabolic rates (SMR) of European and New Zealand short finned-eels do not differ. Since the SMR is defined as the metabolic rate of a resting, fasting fish at a particular temperature (Videler, 1993), it is the sum of all energetic processes occurring during rest, including the response to stress (Sloman et al., 2000). Cortisol is the end product of the Hypothalamic-Pituitary-Interrenal axis in fish, which is activated as a physiological response to stress (Wendelaar-Bonga, 1997). Blood cortisol levels regulate the basic metabolic rate through various processes, including heart beat rates (Davis and Schreck, 1997). Therefore, similar SMRs indicate similarity in stress levels as responses to handling, housing, and transport. Also, it has been shown that swimming exercise suppresses stress and cortisol based stress effects in fish (Milligan, 1996). It therefore can be assumed that observed differences in swimming speeds may not be based on different stress levels or maintenance costs between European and New Zealand short-finned eels. However, a definitive conclusion can only be made by simultaneously measuring blood cortisol levels.

Regardless the COT_min_ or swimming speeds during migration, if the spawning migration can be completed, reproduction successfully accomplished and enough fat incorporated into the eggs for the larvae to survive until they can independently feed, is not to be predicted from the present data for both, European and New Zealand short finned-eels. Additional research will have to be conducted.

### Implications for telemetry studies

The optimal swimming speed (U_opt_) for European eels, i.e., the velocity with the lowest cost of transport (COT_min_) and therefore the presumed migration speed, is similar to previous findings (Palstra et al., 2008; Burgerhout et al., 2011; Methling et al., 2011). According to Tesch (2003), European eels would require a migration speed of 35 km d^−1^ or 0.4 m s^−1^ to reach the postulated spawning ground in the Sargasso Sea within the expected 6 months. Field studies (e.g., Aarestrup et al., 2009), show that eels equipped with an external satellite tag travel at an average migration speeds of 5–25 km d^−1^, i.e., 0.1–0.3 m s^−1^, significantly lower than the required migration or measured optimal swimming speed. However, the suggestion that these eels are impaired by the added drag of the tag and consequently reach lower migration speeds can be dismissed. Various recent experimental studies on the swimming capacity of externally tagged eels (Burgerhout et al., 2011; Methling et al., 2011; Tudorache et al., 2014) showed that U_opt_-values of tagged eels were similar to those of untagged individuals, suggesting that other factors may compensate for the added drag. Indeed, these studies find an increase in COT_min_. It was therefore argued that U_opt_ was traded off by an increase in COT_min_, allowing preserved migration period in order to reach the spawning area in due time. Therefore, as a strategy, tagged animals may try to swim with the same U_opt_, but at an increased COT_min_ (Methling et al., 2011).

Equipping New Zealand short-finned eels with a satellite tag would yield a wealth of information about their spawning migration. Additionally, it could potentially be more rewarding than studying species migrating over longer distances, such as the European eel, since the risk of losing the tagged animal is reduced. However, nothing is known about the migration period of this species. Considering the lower U_crit_ and U_opt_ New Zealand short-fin eel as compared to European eel, but similar energetic values (AMR_crit_ and AMR_opt_, respectively), added drag by a similarly sized tag could increase the associated COT_min_ by factor 3 (Burgerhout et al., 2011). Since the COT_min_ is already higher in New Zealand than in European eels, an increase would come even closer to the measured maximum energetic capacity, expressed as AMR_crit_. As a result, this increased reduction in scope for activity could lead to a faster depletion of fuel reserves in New Zealand short-finned eel, traveling over the same distance as European eel. However, since anguillid eels are assumed to deplete their energy reserves during migration and subsequent reproduction, without refueling (Tesch, 2003), starvation can be a threat to migrating eels. It therefore was previously suggested that one of the factors determining the onset of the spawning migration are energetic reserves in the form of body fat (van den Thillart et al., 2004).

Additional research is necessary to study the effect of external tracking devises on the swimming capacity of New Zealand short-finned eel.

### Methodological implications

U_crit_ is often defined as the maximum prolonged swimming speed using aerobic and anaerobic metabolism (Plaut, 2001; Blake, 2004), i.e., the swimming speed at which both aerobic and anaerobic exhaustion occurs (Lurman et al., 2008). It is therefore an ecologically significant indicator for the migration capacity of a species. However, recent studies have shown that the results of the U_crit_-test depends on a variety of experimental factors unrelated to aerobic or anaerobic swimming capacity, including flume length (Kieffer, 2000; Tudorache et al., 2007; Deslauriers and Kieffer, 2012) or post exercise impingement against the rear grid of the swimming tunnel (Tudorache et al., 2010). Additionally, time interval and velocity increment used during the test have been shown to affect U_crit_ (Farlinger and Beamish, 1977; Farrell, 2007). Farlinger and Beamish (1977) showed that with an increase in velocity increments at a fixed time interval, U_crit_ of largemouth bass reached higher values, and with an increase in time intervals at fixed velocity increments, U_crit_ decreased curvilinearly. Farrell (2007) suggests that the duration of the speed increment is important because of a minimum time-interval needed for cardiorespiratory activity to reach a steady state. Even though heart rate can change quickly, cardiac output, blood pressure, and blood gas tensions can take several minutes to reach a steady state at a new speed increment. This allows the following conclusions: firstly, U_crit_-values are not to be extrapolated to natural conditions, i.e., the U_crit_ only rarely represents the maximum prolonged swimming speed of a freely swimming fish in nature; secondly, in order to apply U_crit_ as an indicator for swimming fitness to compare species or conditions, the U_crit_-test must be performed using the same experimental parameters. In the present study, oxygen consumption rate (ṀO_2_) was measured during 60 min time intervals. These long time intervals were necessary for a reliable measurement of oxygen consumption rate with the set up used (van den Thillart et al., 2004). The U_crit_-test on the other hand, was performed using a time interval of 20 min, in order to compare the results to previous work on swimming eels (Methling et al., 2011; Tudorache et al., 2014), using time intervals of 20 min. Therefore, we chose to measure ṀO_2_ and U_crit_ in two separate tests with different time intervals.

Similar studies on other species suggested a fitting accuracy (*r*^2^) of more than 0.9 for oxygen measurements over time (Schurmann and Steffensen, 1997; Behrens and Steffensen, 2007), while our results are based on an *r*^2^ of 8.9 ± 2.2. This reduced accuracy in data distribution prevented the measurement of Excess Post-Exercise Oxygen consumption (EPOC), an indicator for the anaerobic capacity of swimming fish (Lee et al., 2003; Svendsen et al., 2010). The Blazka-type set up used in this study is unique with an elongated swimming chamber especially designed for anguilliform swimmers (van den Thillart et al., 2004). The disadvantage is that a relatively large water volume produces more background noise in the measurements and the *r*^2^ therefore is reduced. However, since the results in our study compare with those from previous studies obtained in the same (e.g., Palstra et al., 2008; Burgerhout et al., 2011) or other set ups (e.g., Methling et al., 2011), they can be considered valid. Ideally, respirometry studies on anguilliform swimmers should be conducted using a flume combining an elongated swimming chamber with a low water volume to fish body mass ratio. Similarly, background oxygen consumption by aerobic bacteria can be accountable for a large noise signal in the oxygen measurement. Additional oxygen measurement before and after swimming trials can help to eliminate this potential noise source.

Previous studies suggest correcting for the solid blocking effect (Schurmann and Steffensen, 1997; Methling et al., 2011), while other studies (Jones et al., 1974) claim that a correction is not necessary if cross sectional area of the fish is below 10% of that of the swimming tunnel. Correction for solid blocking effect in the present study resulted in increased water velocities of 5.2 ± 1.6%, which was statistically negligible. However, we advise to perform this correction when data are compared to other laboratory or field studies, because the actual swimming speed could be significantly higher.

The exponential equation used in the present study is based on work by Brett (1964) and Webb (1975) and has been used thereafter on a large variety of fish species. Other studies (e.g., Methling et al., 2011) use a power equation, based on hydrodynamic models. However, there are only two constants to derive in an exponential equation, the SMR and the constant c, which is an inversion of U_opt_. Since a power function has three constants to derive, the exponential function is more robust. Also, it is more reliable for making predictions beyond the range of measured values (Korsmeyer et al., 2002). This is particularly important for estimating SMR and U_opt_, crucial values for the interpretation of this study. A power-based equation would tend to overestimate the SMR, because it weighs ṀO_2_-values at higher swimming speeds more heavily than at lower swimming speeds (Roche et al., 2013). Additionally, a power-based model assumes maintenance costs to remain similar over a range of different swimming speeds, which may not be the case (Farrell and Steffensen, 1987). Considering the high variability of ṀO_2_ data at high swimming speeds, and the subsequent unreliability of derived and extrapolated data such as SMR and U_opt_, we chose for the exponential approach. However, whether a model is chosen first and the goodness of fit is calculated subsequently, or the type of model is selected based on goodness of fit, depends on the choice of approach. For a comparative study, the model should be chosen first, in order to calculate how well the data fit the model. For an explorative approach, the model can be chosen based on fit. The present study aimed to compare the swimming physiology of two species; therefore we chose the model first. However, regardless the model chosen for plotting ṀO_2_, our extrapolated values for SMR are similar to those reported by Methling et al. (2011), who used a power-based model, and our U_opt_-values represent those by Methling et al. (2011) and Burgerhout et al. (2011) who used even a polynomial model. These similarities in data suggest validity for a larger array of models.

## Conclusion

This is the first direct experimental comparison of the swimming capacity of two anguillid eel species, the European eel (*A. anguilla*) and the short-finned eel (*A. australis*). As hypothesized, European eels have a higher U_crit_ and U_opt_, and a lower COT_min_ than New Zealand short-finned eels, suggesting higher overall swimming capacity, possibly as an adaptation to a longer migration distance. Swimming and energetic parameters obtained in this study can be used for the design and the evaluation of telemetry studies on New Zealand short-finned eels, from a direct comparison with European eel swimming capacity and energetics.

### Conflict of interest statement

The authors declare that the research was conducted in the absence of any commercial or financial relationships that could be construed as a potential conflict of interest.
